# Comparison of the efficacy of posterior-anterior screws, anterior-posterior screws and a posterior-anterior plate in the fixation of posterior malleolar fractures with a fragment size of ≥ 15 and < 15%

**DOI:** 10.1186/s12891-020-03594-7

**Published:** 2020-08-22

**Authors:** Zheng Wang, Jianbin Sun, Jun Yan, Pengcheng Gao, Hao Zhang, Yong Yang, Qunhua Jin

**Affiliations:** Department of Orthopaedic, General Hospital of Ningxia Medical University, Yinchuan, 750004 Ningxia China

**Keywords:** Posterior malleolar fracture, Cannulated screws, Reconstruction plate, Posterolateral approach

## Abstract

**Background:**

Different fixation methods have been used to treat posterior malleolar fractures (PMFs), but the clinical efficacy of different fixation methods in the treatment of PMF with different fragmentation has rarely been reported. The purpose of this study was to investigate the efficacy of posterior-anterior (PA), anterior-posterior (AP) screws and PA plate in the fixation of PMFs with a fragment size of ≥15 and < 15%.

**Methods:**

This is a retrospective study of the clinical data of 243 patients with a unilateral ankle fracture involving the posterior malleolar ankle fracture. All patients were divided into two groups based on their fragment size, ≥15% (*n* = 136) and < 15% (*n* = 107). After reduction of PMF under direct vision via a posterolateral approach, posterior-anterior (PA), anterior-posterior (AP) screws and PA plate were used for fixation of PMF in the two groups. Briefly, for fixation of PMF with PA screw, two to three 3.5-mm (Depuy Synthes, Switzerland) cannulated screws were placed from the posterior to anterior direction; for fixation with PA plate, a 3.5-mm reconstruction plate (Depuy Synthes, Switzerland) was placed from the posterior to anterior direction, and for fixation of PMF with an AP screw, two to three 3.5-mm screws were placed from the anterior to posterior direction. All patients were followed up at 1, 3, 6, and 12 months after surgery and thereafter at 6-month intervals. The primary outcomes were AOFAS and ROM, which were recorded at the final follow-up.

**Results:**

The average follow-up time for all patients was 18.9 months (range 12–36 months), and all fractures healed. In fragment size ≥15% group, the average AOFAS score of patients treated with PA, AP screws and posterior plate were 91.5, 91.8, and 90.8, respectively, and the average limited ankle-dorsiflexion ROM was 5.0 °, 5.4 ° and 5.6°, respectively, at the last follow-up, there was no significant difference between the three fixation methods in terms of AOFAS scores and ankle ROM (*P* > 0.05). In fragment size < 15% group, the average AOFAS score of patients treated with PA, AP screws and posterior plate were 92.3, 91.9, and 84.1, respectively, the average limited ankle-dorsiflexion ROM were 5.1 °, 4.7 °, and 6.3 °, respectively, at the last follow-up. There were statistically significant differences in AOFAS scores and ankle ROM between posterior plate fixation and PA, AP screw fixation (*P* < 0.05); while no significant difference was found between PA and AP screw fixation (*P* > 0.05).

**Conclusion:**

For PMFs with fragment size ≥15%, there was no significant difference in the outcomes between the three fixation methods. For PMF with fragmentation < 15%, the PA and AP screws both provided good fixation.

## Background

The posterior malleolus is a component of the distal tibiofibular complex and is involved in maintaining the stability of the ankle joint [1]. Posterior malleolar fractures (PMFs) account for about 7 to 44% of all ankle fractures [2]. At present, the indications for surgical treatment of PMF are still controversial. It is widely accepted that if the PMF involves more than 25% of the articular surface estimated from the lateral X-rays, and if the fragment is displaced more than 2 mm, surgical treatment is required [3, 4]. Previous studies have confirmed that PMFs involving more than 25% of the articular surface may lead to an increased incidence of post-traumatic osteoarthritis [5, 6]. However, Langenhuijsen [7] suggested that small PMF fragments also require surgical reduction and fixation. If the fragments involve 10% of the articular surface of the tibia, anatomical reduction and internal fixation are indicated to restore the congruency of distal tibiofibular syndesmosis, the tension of the posteroinferior tibiofibular ligament, and the stability of the distal tibiofibular syndesmosis. Owing to limitations of X-ray examination, lateral X-rays of the ankle cannot accurately show the size of PMF fragments [8]. Leung K. H, et al. [9] believed radiography alone is not adequate for surgical planning for ankle fractures. More accurate imaging tools such as CT are needed to enable a more accurate diagnosis and surgical planning. Haraguchi et al. [10] suggested the use of CT to reliably estimate the fragmentation percentage. Yi [11] accurately determined the size of PMF fragments (≥15 and < 15%) according to the fragment area ratio (FAR) measured using CT cross-section images. Another study showed that the treatment satisfaction of patients with ankle fractures with PMF were worse than those without [12]. Therefore, to prevent poor function of the ankle joint and the occurrence of post-traumatic arthritis, more attention should be paid to PMF [13]. At present, the reduction of PMF directly through a posterior approach is the most effective technique [14]. Different fixation methods have been used to treat PMF, but the preferred fixation method remains controversial. One study showed that the use of a posterolateral plate is superior to posterior lag screws for fixation of PMF [15]. However, another clinical study [16] revealed that no significant outcome differences were observed among patients with different fixations. The clinical efficacy of different fixation methods in the treatment of PMF with different fragmentation has rarely been reported. Therefore,In the present study, patients with PMF were divided into ≥15 and < 15% fragmentation groups according to the FAR value measured from CT cross-sectional images, and used a posterior-anterior (PA) cannulated screw, anterior-posterior (AP) lag screw, and posterior plate to fix the PMF, and compare the difference in the treatment outcomes between these three fixation methods.

## Methods

### Subjects

We conducted a retrospective study of 435 patients with unilateral ankle fracture involving the posterior malleolar ankle fracture who were admitted to our hospital from January 2015 to September 2018. The inclusion criteria were: (1) aged between 18 and 65 years; (2) patients with unilateral ankle fractures involving the the posterior malleolar ankle fracture and their contralateral side is normal; (3) all fractures are classified according to Lauge-Hansen classification, supination external rotation IV-fracture and pronation-external rotation type-IV fracture were included. The exclusion criteria were: (1) patients with open fractures, Pilon fractures, pathological fractures, old fractures, or fractures of other parts of the ipsilateral lower limb; (2) patients who had severely degenerative or inflammatory ankle arthritis; (3) patients who received conservative treatment for PMF.

**Fig. 1:**
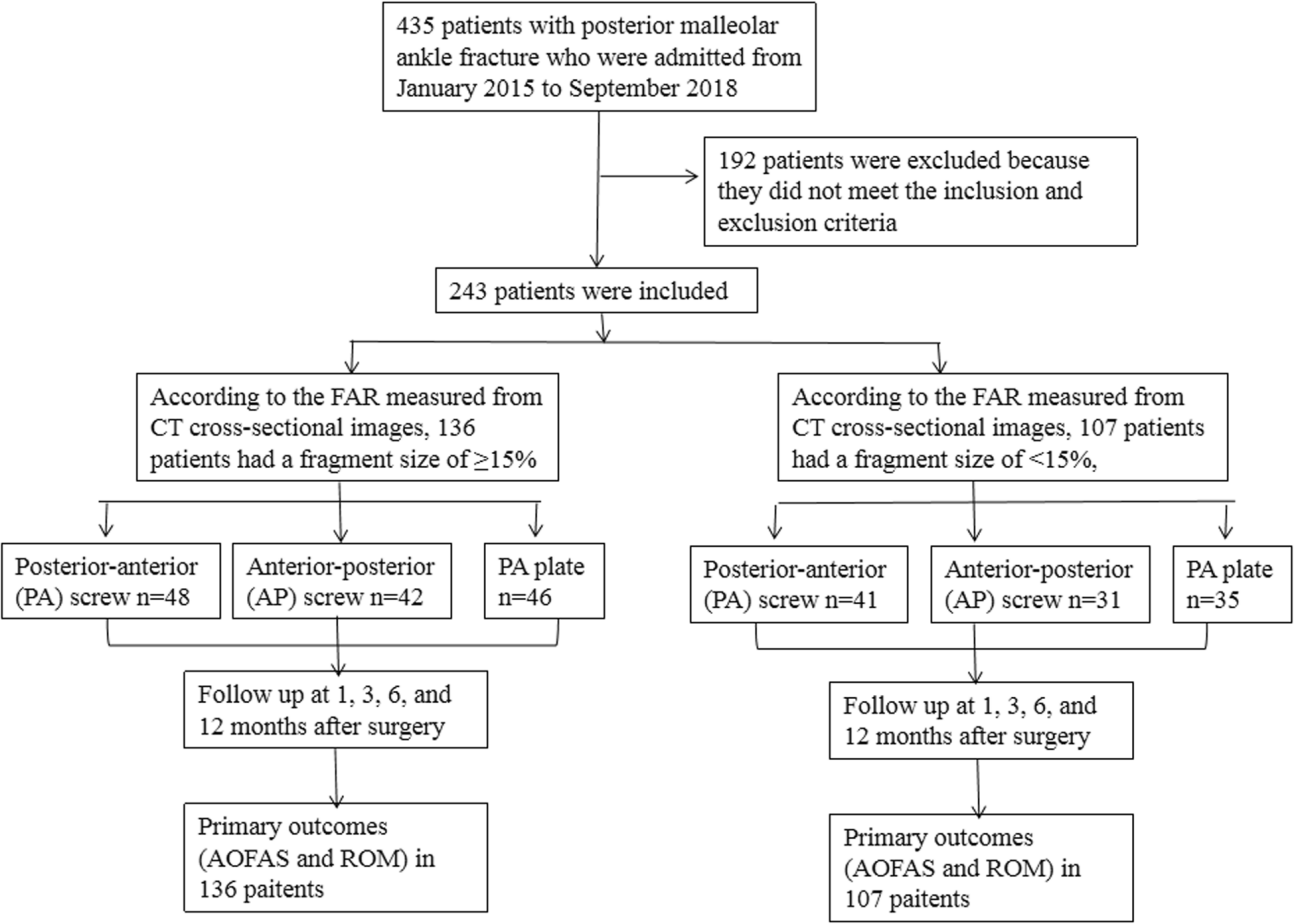
Flow chart of inclusion and exclusion criteria of patients with posterior malleolar fracture

Two hundred forty-three patients met the inclusion and exclusion criteria and were enrolled in this study. According to the FAR measured from CT cross-sectional images, all patients were divided into fragmentation groups, ≥15% (*n* = 136) and < 15% (*n* = 107), and each group was subdivided into PA screw, AP screw, and PA plate subgroups based on the method of fixation. Figure 1 showed the flow chart for inclusion and exclusion of patients. This study was approved by the Ethics Committee of General Hospital of Ningxia Medical University, China.

### Surgical methods

All operations were performed by a single orthopedic trauma surgeon with more than 5 years of trauma and ankle surgical experience. Three fixation methods were randomly performed on these patients according to patients’ wishes. Under general anesthesia, patients were placed in a floating position, and the air pressure tourniquet was placed on the upper thigh of the limb. Reduction and fixation were performed in order, i.e. reduction and fixation of first the lateral malleolus, the posterior malleolus, then the medial malleolus, and finally stabilization of the distal tibiofibular syndesmosis. When reduction and fixation of the lateral malleolus and posterior malleolus was complete, the patient was placed in the lateral position. The posterolateral approach was selected and reduction under direct vision was performed. The skin incision was made at the midline between the posterior edge of the lateral malleolus and the Achilles tendon, with the distal end extended to the tip of the lateral malleolus. After the skin incision, the subcutaneous tissue and deep fascia were exposed with attention paid to protect the small saphenous vein and sural nerve. The deep fascia was cut longitudinally to expose the peroneal tendon. The peroneal tendon was pulled laterally, and the lateral malleolus fracture was reduced and fixed. Then, the posterior malleolar fragment of the distal tibia were exposed along the flexor hallucis longus and peroneus longus. During the operation, care was taken to protect the peroneal artery, posteroinferior tibiofibular ligaments, and periosteum on the fracture fragments. After clearing the fracture site, the PMF was reduced under direct vision. Kirschner wires were used for temporary fixation, two to three 3.5-mm (Depuy Synthes, Switzerland) cannulated screws were placed from the posterior to anterior direction.

Fixation with the plate required extensive dissection of the flexor hallucis longus and peroneus longus, the fragment was reduced under direct vision and a 3.5-mm reconstruction plate (Depuy Synthes, Switzerland) was placed from the posterior to anterior direction. After the lateral malleolus and posterior malleolus were reduced and fixed, the patient was placed in the supine position, a curved incision was made on the anteromedial aspect of the medial malleolus, the medial malleolar fracture was reduced under direct vision and fixed with two 3.5-mm screws (Depuy Synthes, Switzerland). For fixation of PMF with an AP screw, PMF was reduced under direct vision via a posterolateral approach, and temporarily fixed with a K-wire. Then, the patient was placed in the supine position. Through the curved incision on the anteromedial aspect of the medial malleolus, two to three 3.5-mm screws were placed from the anterior to posterior direction to fix the PMF, and fix the medial malleolus. A cotton test was performed during the operation to determine the stability of the tibiofibular syndesmosis. If instability was present, a 3.5-mm lag screw was used to stabilize the syndesmosis.

### Postoperative management

After surgery, all patients in each group received the same rehabilitation treatment (treatments routinely conducted to prevent infection, decrease swelling, and prevent deep vein thrombosis in the affected limb). After 24 h of surgery, the drainage tube was removed, then patients were allowed to perform active and passive functional exercises. X-rays were performed to assess fracture healing. For patients who received fixation for separation of distal tibiofibular syndesmosis, the lag screws were removed 12 weeks after surgery. All patients were allowed to bear full weight at 3 months postoperative.

### Outcomes evaluation

All patients were followed up at 1, 3, 6, and 12 months after surgery and thereafter at 6-month intervals. During the follow-up period, anterioposterior and lateral X-rays were taken to observe the status of the fracture healing. During the final follow up, functional outcomes were assessed with the American Orthopaedic Foot and Ankle Society (AOFAS) score [17].

The range of motion (ROM) of the ankle was measured, and differences in the ankle dorsiflexion of the affected and unaffected side was calculated and consider as limited ankle-dorsiflexion ROM [13].

### Statistical analysis

Statistical analyses were performed using SPSS 22.2 software. Comparisons were made between the three subgroups (PA screw, AP screw, and PA plate) within fragmentation groups of ≥15 and < 15%.

Th age of the three subgroups within each group presented normal distribution with equal variances, and were expressed as mean ± standard deviation (SD). The differences between the three subgroups within the groups were determined using one-way analysis of variance (ANOVA). If there was a statistically significant difference, multiple comparisons were performed using the LSD post hoc test.

The time from injury to surgery, follow-up time, and AOFAS score of the three subgroups presented non-normal distribution with unequal variances, and were expressed as a median (P25, P75). The differences between the three subgroups within the ≥15 and < 15% fragmentation groups were compared using the Kruskal-Wallis H test. If there was a statistically significant difference between the three subgroups within the groups, post hoc multiple comparisons were performed and the *P* values were corrected for multiple comparisons.

We compared differences in sex, the Lauge-Hansen classification, and mechanisms of injury between the three subgroups within the ≥15 and < 15% fragmentation groups using a χ^2^ test. If there were a statistically significant difference between three subgroups within the groups, a partitioned chi-square test was used for pairwise comparisons, and significance level α was adjusted and set at α = 0.05.

## Results

### Demographic characteristics of the patients

Among these 243 patients, 120 were male and 123 were female, there were 119 ankle sprains, 68 due to a high falling injury, and 56 due to traffic injuries. According to Lauge-Hansen classification, there were 117 cases of supination external rotation IV-fracture and 126 cases of pronation-external rotation type-IV fracture. According to the FAR measured from CT cross-sectional images, 136 cases had a fragment size of ≥15%, 107 patients had a fragment size of < 15%. Among these 136 patients with a fragment size of ≥15%, 48 patients were treated with PA screws, 42 patients were treated with AP screws, and 46 patients were treated with a PA plate. Among these 107 cases with a fragment size of < 15%, 41 patients were treated with PA screws, 31 patients were treated with AP screws, 35 patients were treated with a PA plate. There were no significant differences between patients in the groups with respect to age, sex, Lauge-Hansen classification, mechanisms of injury, time from injury to surgery, or follow-up time (Tables 1 and 2).

**Table 1:** Demographics of patients with a PMF fragment size ≥15%

	Posterior-anterior (PA) screw (*n* = 48)	Anterior-posterior (AP) screw (*n* = 42)	Posterior plate (*n* = 46)	χ2/*F*	*P*
Sex					
Male	25	20	21	0.409	0.815
Female	23	22	25		
Lange-Hansen classification					
Supination external rotation	26	19	20	1.235	0.539
Pronation-external rotation	22	23	26		
Mechanisms of injury					
Low-energy	22	22	24	0.516	0.773
High-energy	26	20	22		
Age (Year)	46.6 ± 12.3	44.2 ± 14.1	42.1 ± 11.9	1.478	0.232
Time from injury to surgery (Day)	6.8 [6.0,8.7]	6.6 [5.8,8.4]	7.1 [5.6,8.1]	0.703	0.704
Follow-up time (Month)	18.2 [14.5,23.6]	17.7 [14.4,22.3]	17.1 [14.5,19.8]	0.856	0.652

**Table 2:** Demographics of patients with a PMF fragment size < 15%

	Posterior-anterior (PA) screw (*n* = 41)	Anterior-posterior (AP) screw (*n* = 31)	Posterior plate (*n* = 35)	*χ2*/*F*	*P*
Sex					
Male	21	17	16	0.563	0.755
Female	20	14	19		
Lange-Hansen classification					
Supination external rotation	22	13	17	0.971	0.615
Pronation-external rotation	19	18	18		
Mechanisms of injury					
Low-energy	19	14	18	0.305	0.858
High-energy	22	17	17		
Age (Year)	43.8 ± 11.8	45.8 ± 13.4	43.2 ± 11.9	0.382	0.683
Time from injury to surgery (Day)	8.6 [7.6,11.4]	8.8 [8.0,10.0]	8.5 [8.0,9.8]	0.412	0.814
Follow-up time (Month)	17.5 [13.9,24.2]	17.7 [15.1,20.9]	17.9 [13.9,22.7]	0.315	0.854

**Fig. 2:**
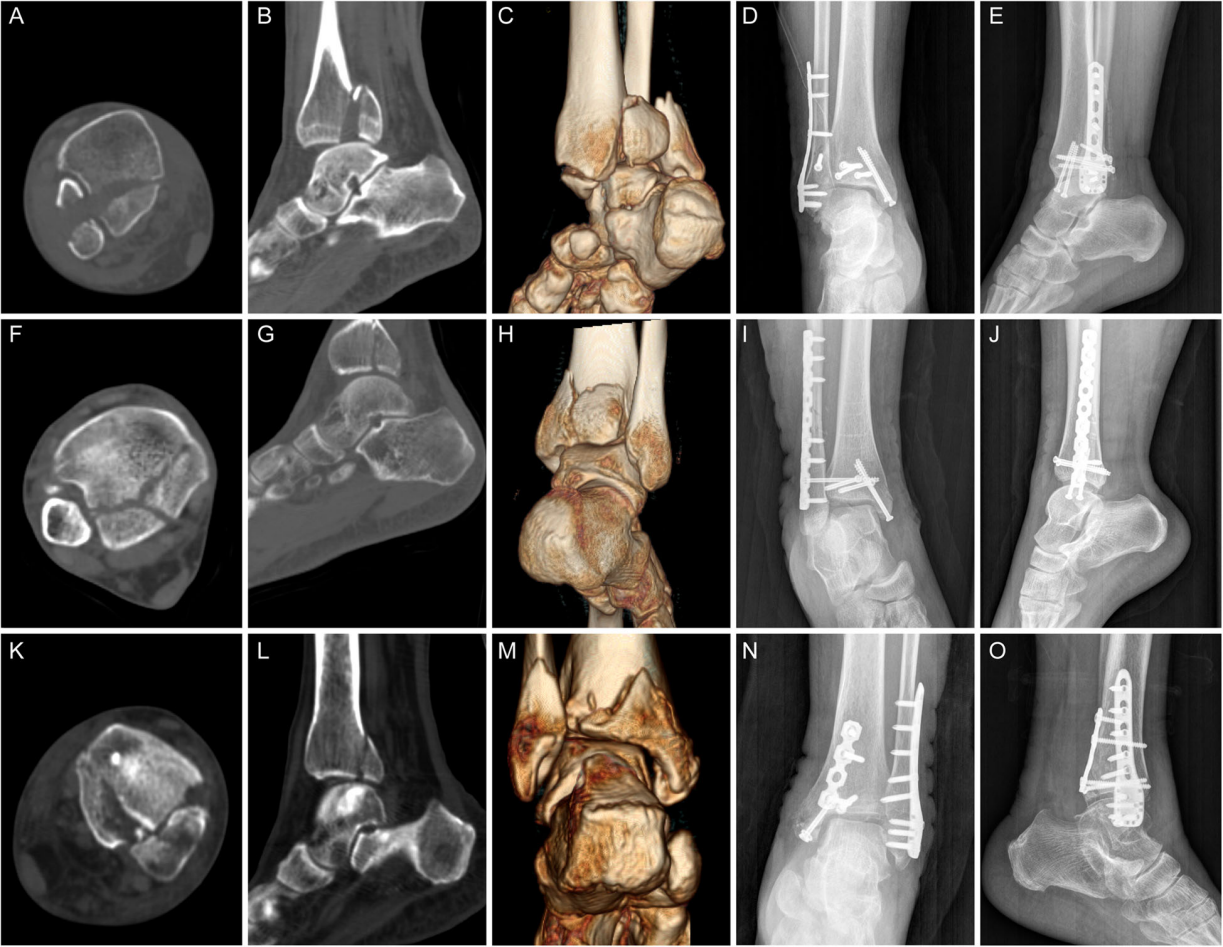
CT and X-ray images of patients who had posterior malleolar fracture with fragment size of ≥15%. **a**, **b**, **c**, **f**, **g**, **h**, **k**, **l**, **m**: preoperative CT images; **d**, **e**: Postoperative X-ray of a patient showing fixation with a PA screw; **i**, **j**: postoperative X-ray of a patient showing fixation with a AP screw; **n**, **o**: postoperative X-ray of a patient showing fixation with a posterior plate

**Fig. 3:**
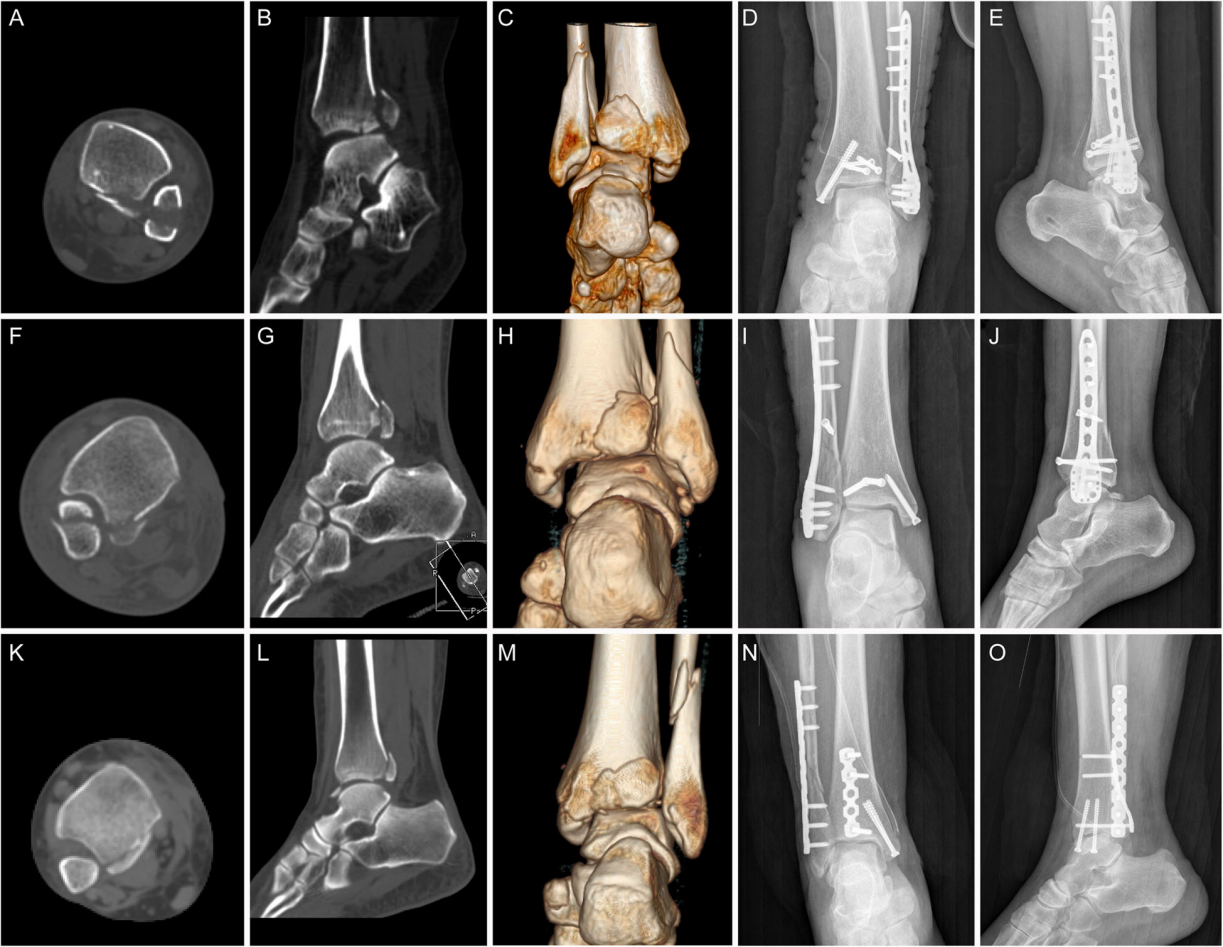
CT and X-ray images of patients who had posterior malleolar fracture with fragment size of < 15%. **a**, **b**, **c**, **f**, **g**, **h**, **k**, **l**, **m**: preoperative CT images; **d**, **e**: Postoperative X-ray of a patient showing fixation with a PA screw; **i**, **j**: postoperative X-ray of a patient showing fixation with a AP screw; **n**, **o**: postoperative X-ray of a patient showing fixation with a posterior plate

All patients were followed up for 12–36 months, with an average of 18.9 months. Bone healing was achieved in all patients, with no fracture nonunion, loosening or rupture of the implant, or fracture displacement observed (Figs. 2 and 3). Two patients in the fragmentation ≥15% group and one patient in the fragmentation < 15% group developed superficial infections after surgery, and were treated successfully with local dressing changes and oral antibiotic therapy. No deep infections occurred. No patient had complications related to sensory loss of the sural nerve.

### AOFAS score

In the fragmentation ≥15% group, the average AOFAS score of patients treated with a PA screw, AP screw, and PA plate were 91.5, 91.8, and 90.8, respectively, at the final follow-up and no statistical difference was found between the three fixation methods (*P* > 0.05). In the fragmentation < 15% group, the average AOFAS score of patients treated with a PA screw, AP screw, and PA plate were 92.3, 91.9, and 84.1, respectively, at the final follow-up. There were statistically significant differences in the AOFAS score between the PA plate fixation and screw fixations (*P* < 0.05), while there was no significant difference between the PA and AP screw fixation (*P* > 0.05) (Tables 3 and 4).

**Table 3:** Outcomes of patients with a PMF fragment size ≥15%

	Posterior-anterior (PA) screw (*n* = 48)	Anterior-posterior (AP) screw (*n* = 42)	Posterior plate (*n* = 46)	χ2	*P*
AOFAS score	91.5 [87.6,96.2]	91.8 [89.2,95.1]	90.8 [88.5,93.2]	1.688	0.430
Dorsiflexion restriction	5.0 [3.9,7.8]	5.4 [4.0,7.9]	5.6 [4.7,6.8]	0.929	0.628

**Table 4:** Outcomes of patients with a PMF fragment size < 15%

	Posterior-anterior (PA) screw(*n* = 41)	Anterior-posterior (AP) screw(*n* = 31)	Posterior plate (*n* = 35)	*χ2*	*P*
AOFAS score	92.3 [88.0,94.9]	91.9 [89.7,94.0]	84.1 [80.5,86.5] **^△△^	39.552	0.000
Dorsiflexion restriction	5.1 [3.8,6.1]	4.7 [3.7,6.6]	6.3 [5.1,7.9] *^△△^	13.282	0.001

*P < 0.05, **P < 0.01, vs posterior-anterior (PA) screw group; ^△^*P* < 0.05, ^△△^*P* < 0.01, vs anterior-posterior (AP) screw group

### The limited ankle-dorsiflexion ROM

In the fragmentation ≥15% group, the average limited ankle-dorsiflexion ROM for patients treated with the PA screw, AP screw, and PA plate had the mean limited dorsiflexion of 5.0 °, 5.4 °, and 5.6 °, respectively, at the final follow-up, there was no significant difference between the three fixation methods. In the fragmentation < 15% group, the average ROM at the final follow-up for patients treated with the PA screw, AP screw, and PA plate were 5.1 °, 4.7 °, and 6.3 °, respectively. There were statistically significant differences between the PA plate fixation and screw fixations (*P* < 0.05), while no significant difference was found between the PA and AP screw fixation (*P* > 0.05, Tables 3 and 4).

## Discussion

The goal of the surgical treatment of ankle fractures is to restore the normal anatomical morphology of the ankle, maintain joint stability, and reach maximum functional recovery. Unlike the treatment for medial and lateral malleolar fractures, the surgical indications and surgical fixation methods for PMF are inconclusive [18]. Therefore, in this study, we investigated the efficacy of posterior-anterior (PA), anterior-posterior (AP) screws and PA plate in the fixation of PMFs with a fragment size of ≥15 and < 15%.

In previous studies, PMFs with small displacement were often reduced indirectly and fixed with screws using the anterior to posterior approach [19–21], which can avoid delayed wound healing, soft tissue adhesion and iatrogenic sural nerve damage caused by soft tissue dissection. However, due to the interposition of soft tissue or loose osseous fragments in the PMF gaps, it is difficult for indirect reduction to achieve anatomical reduction, and it is technically difficult to fix small or comminuted fragments [22]. In recent years, more attention has been paid to the importance of anatomical reduction and internal fixation of PMF [23–26]. Anatomical reduction and fixation of posterior ankle fractures can be achieved under direct vision using a posterolateral approach, which is highly accepted [14, 22, 27]. In this study, we chose the posterolateral approach to reduce posterior ankle fractures under direct vision. When the fragment size was ≥15%, there was no statistical difference in the AOFAS score, with limited dorsiflexion ROM between the PA screw and PA plate fixations, which is similar to the results of a previous study [16]. Our findings suggested that the fixation of PMF with a fragment size of ≥15% using either a PA screw or PA plate can both achieve satisfactory outcomes. Unlike previous studies of fixation of PMF using AP screws with indirect reduction [19–21], in this study, when fixation of PMF was with an AP screw, we also performed anatomical reduction of PMF under direct vision, and placed the AP screw through an incision on the anteromedial aspect of the medial malleolus to fix the fracture. Our results demonstrated that with a fragment size ≥15%, no statistical differences in the outcomes after AP plate were observed compared with the AP plate or PA screw fixation.

Anwar et al. [28] performed a biomechanical study of different fixation methods for fixation of PMF using finite element analysis and found that fixation of a posterior ankle fracture using a PA plate or PA screw can achieve better clinical results than AP screw fixation, and the plate had higher fixation strength. The reason for the difference between our results and the above previous study may be that in our study, the posterolateral approach is selected, fracture reduction is more accurate under direct vision, and the fixation is also performed under direct vision, which can avoid the poor outcomes caused by a lag screw that is too short or in a poor position, and improve the efficacy of the fixation. Additionally, AP screw fixation does not increase secondary injuries and can be removed easily. Therefore, although the fixation strength of the AP screw is not as high as the PA plate, the AP screw fixation with direct reduction via a posterolateral approach may also be effective for fixation of PMF with a fragment size of ≥15%.

Whether there is any need to perform surgical fixation for PMF with low fragmentation remains inconclusive. Langenhuijsen et al. [7] emphasized the importance of the quality of the posterior malleolus reduction, and advocated that the joint congruity, not the fragment size, is the main factor determining the need for fracture fixation. Verhage et al. [29] suggested to correct intra-articular step-off of intraarticular posterior malleolar fragments to reduce the risk of developing osteoarthritis. Tosun et al. [30] showed that the fixation of PMF is closely related to the successful radiological and functional outcomes of trimalleolar fracture. After PMF is fixed, transyndesmal screw fixation may not be required. For these reasons, the authors believe that PMF with all fragment sizes should be fixed. Gardner et al. [24] performed a study investigating the role of the fixation of PMF in the stability of the distal tibiofibular syndesmosis, they concluded that the fixation of PMF provided greater syndesmotic stability than the fixation of the distal tibiofibular syndesmosis alone. Therefore, in this study, the same surgical treatments were performed for patients who had a fragment size of < 15% and ≥ 15%.

Interestingly, our results showed that when the fragment size was < 15%, there were statistically significant differences in the AOFAS scores and limited ankle-dorsiflexion ROM between the posterior plate fixation and screw fixations. The results indicated that the PA screw or AP screw fixation may be more effective than PA plate fixation for PMF with a fragment size of < 15%. The reason may be that when the fragments are small and located at the distal end of the tibia, the plate needs to be placed at the distal end of the tibia to obtain effective fixation. However, when the plate is placed close to the distal end of the tibia, the very distal part of the plate may protrude, and unlike the screw, the plate cannot be countersunk, which can stimulate flexor hallucis longus, cause the formation of serious adhesions [28], and affect postoperative outcomes. These problems may also exist when treating PMFs with a fragment size of ≥15%, but when the fragments are larger, the plate can be placed slightly more proximal to the tibia, which can reduce the protrusion of the very distal part of the plate, thereby reducing the postoperative adhesions of the flexor hallucis longus. Therefore, different outcomes were obtained during treatment of PMF with fragment size of ≥15 and < 15% using the PA plate. However, further investigations are needed to verify these results.

### Limitations

There are some limitations in our study that need to be considered. In this study, the fragment size was assessed according to the ratio of the fragment area to the total cross-sectional area of the tibial plafond measured based on axial CT scans, which is not assessed according to the anteroposterior (sagittal) diameter of the tibia measured using X-ray. The indication of ORIF for PMF is based on the fragment sizes measured on X-ray. The fragment sizes can be determined more accurately based on axial CT scans, but it also affects the evaluation of the indication of surgical treatment. In addition, we did not grade and compare the status of radiographic osteoarthritis. Theoretically, anatomical reduction and rigid fixation of PMF can restrict the talus, stabilize the syndesmosis, and reduce the risk of post-traumatic osteoarthritis [21]. Furthermore, we did not compare intraoperative indicators, such as surgical time, surgical bleeding volume, and radiation time between the three fixation methods for treatment of PMF with fragment size of ≥15 and < 15%. Further prospective randomized controlled studies with larger sample sizes are needed to confirm these findings.

## Conclusions

Each fixation method for the treatment of ankle fractures has its own advantages and disadvantages. Our findings suggested that there was no statistical difference in AOFAS scores or limited ankle-dorsiflexion ROM among patients who had a fragment size of ≥15% regardless of the fixation type of the posterior malleolus. AP screws are easy to use and remove, and a posterior plate is biomechanically the most stable method for fixation of a PMF. For patients who had a fragment size of < 15%, both PA and AP screws provide good fixation, cause less surgical trauma, and promote postoperative functional recovery.

## Data Availability

The datasets used and/or analyzed during the current study available from the corresponding author on reasonable request.

## Abbreviations

PMFs: Posterior malleolar fractures
PA: Posterior-anterior
AP: Anterior-posterior
FAR: Fragment area ratio
SD: Standard deviation
ANOVA: One-way analysis of variance
ROM: Range of motion
AOFAS score: American Orthopaedic Foot and Ankle Society score
